# Survival Outcomes of Adjuvant Nivolumab after Neoadjuvant Chemotherapy in Esophageal Squamous Cell Carcinoma

**DOI:** 10.5761/atcs.oa.26-00107

**Published:** 2026-07-28

**Authors:** Ruihan Zeng, Shinji Mine, Motomi Nasu, Takashi Hashimoto, Rie Makuuchi, Asako Ozaki, Yutaro Yoshimoto, Takehiro Watanabe, Shotaro Takai, Tetsu Fukunaga

**Affiliations:** Department of Esophageal and Gastroenterological Surgery, Juntendo University, Tokyo, Japan

**Keywords:** nivolumab, esophageal squamous cell carcinoma, disease-free survival, overall survival, immune-related adverse event

## Abstract

**Purpose:**

Given the limited Japanese real-world evidence, this study aimed to evaluate the survival and safety of adjuvant nivolumab after neoadjuvant chemotherapy in patients with an incomplete pathological response to esophageal squamous cell carcinoma (ESCC).

**Method:**

This single-center retrospective study included 105 of 259 patients with ESCC who had an incomplete pathological response after neoadjuvant chemotherapy and esophagectomy. Recurrence patterns and immune-related adverse events (irAEs) were analyzed, and disease-free survival (DFS) and overall survival (OS) were compared between the nivolumab and non-nivolumab groups before and after propensity score matching (PSM).

**Result:**

Among 105 patients, 17 received adjuvant nivolumab. Median follow-up was 26.8 months; recurrence occurred in 33 patients (31.4%), and irAEs occurred in 2 nivolumab-treated patients (11.8%). Before PSM, OS and DFS were not significantly different between 2 groups. After PSM, OS remained nonsignificant (P = 0.202), whereas DFS showed a favorable trend toward improvement in the nivolumab group, with 1-year DFS rates of 75.0% versus 68.8% and 3-year DFS rates of 68.2% versus 19.3%, respectively (P = 0.067). Nivolumab was not an independent prognostic factor, although exploratory high-risk subgroups showed favorable DFS trends.

**Conclusion:**

Adjuvant nivolumab showed a nonsignificant favorable trend in DFS after PSM, with late curve separation favoring the nivolumab group and manageable irAEs.

## Introduction

Esophageal squamous cell carcinoma (ESCC) remains one of the most aggressive malignancies of the digestive tract, particularly prevalent in East Asia.^1)^ Despite advances in multimodal therapy, including neoadjuvant chemoradiotherapy (nCRT) or chemotherapy followed by radical esophagectomy, the prognosis of patients with ESCC remains unsatisfactory.^2)^ A significant proportion of patients show incomplete pathological response after preoperative treatment, characterized by the presence of residual viable tumor cells in resected specimens.^3)^ These patients are at high risk of postoperative recurrence and metastasis, highlighting the urgent need for more effective adjuvant strategies to improve long-term outcomes.

Programmed cell death-1 (PD-1) immune checkpoint blockade has revolutionized the treatment landscape of multiple solid tumors, including esophageal cancer.^4)^ Nivolumab, a fully human IgG4 monoclonal antibody targeting PD-1, has demonstrated durable antitumor efficacy and manageable safety profiles across various diseases.^2,5)^ The phase III CheckMate 577 trial provided the first evidence that adjuvant nivolumab significantly prolongs disease-free survival (DFS) compared with placebo in patients with resected esophageal or gastroesophageal junction cancer who had residual pathological disease after nCRT.^6)^ In the ESCC subgroup, the median DFS reached 29.7 months with nivolumab versus 11.0 months with placebo (hazard ratio [HR] = 0.61; 95% confidence interval [CI] 0.42–0.88).^2)^ Based on these results, adjuvant nivolumab has been covered by Japanese health insurance since 2021 and other international guidelines as the standard of care for patients with an incomplete pathological response after nCRT.^7)^

However, several important clinical questions remain unresolved. In contrast to Western practice, where nCRT is widely adopted as part of trimodality therapy,^3)^ Japan predominantly employs neoadjuvant chemotherapy followed by esophagectomy for resectable cStage II–III ESCC.^8,9)^ Consequently, the clinical value of adjuvant nivolumab after neoadjuvant chemotherapy alone—the more common perioperative context in Japan—remains incompletely defined, motivating real-world evaluations in Japanese cohorts.^9,10)^

Therefore, a deeper investigation into the efficacy and survival outcomes of adjuvant nivolumab in ESCC patients with an incomplete pathological response after neoadjuvant chemotherapy is of great clinical relevance. Clarifying its impact on postoperative adverse events (AEs), recurrence patterns, and DFS will provide critical evidence to optimize perioperative management and guide individualized immunotherapy strategies for ESCC.

## Materials and Method

### Patients

This retrospective study included patients diagnosed with ESCC who underwent curative-intent esophagectomy following neoadjuvant chemotherapy at our institution between November 2021 and March 2025. Follow-up for recurrence and survival outcomes was updated through the data cut-off date of March 2026. Eligible patients met the following criteria: (1) histologically confirmed ESCC; (2) received neoadjuvant chemotherapy; (3) residual pathological disease (non-pathological complete response); and (4) underwent curative-intent radical esophagectomy following neoadjuvant chemotherapy. Patients were excluded if they had (1) undergone non-curative resection, including R1 (microscopically positive margin) or R2 (macroscopically residual disease) resection, or other palliative procedures, given the treatment pathway^11,12)^; (2) a history of other malignancies within 5 years; (3) achieved pathological complete response (pCR); (4) undergone nCRT; (5) received postoperative adjuvant therapy other than nivolumab; (6) received immune checkpoint inhibitors before surgery; or (7) insufficient clinical or follow-up data.

The medical records of patients were reviewed to extract data regarding patient characteristics, neoadjuvant chemotherapy regimens, surgical procedures, clinicopathological findings, and postoperative outcomes. Esophagoscopy, ultrasonography, computed tomography (CT), and positron emission tomography CT (PET-CT) were performed to determine the clinical stage and recurrence. The Eighth Edition of the “Tumor,” “Nodes,” “Metastases” (TNM) classification system developed by the Union for International Cancer Control was used to classify the clinical and histopathological findings.^13,14)^ Postoperative complications were evaluated according to the Clavien–Dindo classification.^15)^ Events classified as Clavien–Dindo grade II or higher were considered positive postoperative complications, whereas recurrent laryngeal nerve palsy was considered positive when classified as Clavien–Dindo grade I or higher.

### Neoadjuvant chemotherapy

Neoadjuvant chemotherapy regimens were determined by a multidisciplinary tumor board based on clinical stage and patient condition. Based on the Japanese guidelines,^7,16)^ the standard neoadjuvant chemotherapy for esophageal cancer at our institute utilizes both doublet regimen and triplet regimen since 2022. For patients with clinically resectable, locally advanced (stage II–III) ESCC who were considered suitable for intensive triplet chemotherapy received 2 or 3 cycles of DCF: docetaxel, cisplatin 70 mg/m^2^ day 1, and 5-FU 750 mg/m^2^ day 1 to 5.^17)^ Double chemotherapy regimen was selected before the introduction of DCF, and thereafter, it was still chosen depending on the patient’s status, age, and other comorbidities. They comprised 2 cycles of cisplatin and 5-fluorouracil: cisplatin 80 mg/m^2^ day 1, and 5-fluorouracil 800 mg/m^2^ day 1 to 5.^8)^ Surgical resection was performed approximately within 6 weeks after the completion of the last cycle of treatment.

### Surgery

We performed subtotal esophagectomy via a right thoracoscopic or robot-assisted operation with lymph node dissection of the cervical para-esophageal (including supra-clavicular lymph nodes as indicated by the lesion’s condition), thoracic, and abdominal areas.^18)^ Postoperative complications (anastomotic leak, anastomotic stricture, pneumonia, recurrent laryngeal nerve palsy, chylothorax, and reoperation) were recorded as binary variables (yes/no) during the index hospitalization.

### Adjuvant nivolumab

Patients who presented with an incomplete pathological response after neoadjuvant chemotherapy were eligible for adjuvant nivolumab treatment, and they were asked whether they wanted to receive adjuvant treatment or not. Furthermore, some of them were enrolled in a randomized controlled trial, which compared the adjuvant strategy, and were chosen as the nivolumab group (JCOG2206; jRCTs031230219).^19)^ If they chose to receive adjuvant treatment, they were given adjuvant nivolumab therapy (240 mg every 2 weeks or 480 mg every 4 weeks for 1 year), following the protocol of the CheckMate 577 trial in late 2021. Nivolumab was administered intravenously to patients who provided consent. Treatment continued until recurrence, the incidence of severe AEs, or patient-requested discontinuation. AEs observed during the administration and follow-up periods were graded according to the National Cancer Institute Common Terminology Criteria for Adverse Events (CTCAE), version 5.0.^20)^ The criteria for treatment interruption included the incidence of grade 2 or higher nivolumab-related AEs, grade 3 or higher skin reactions, fatigue, hematologic abnormalities, or any investigator-determined reason. Immune-related adverse events (irAEs) were managed according to published guidelines.^21)^ The specific details about irAEs will be presented one by one.

### Evaluation of tumor histopathological response

According to the Japanese Classification of Esophageal Cancer (12th edition), the tumor regression grade was assessed in surgical samples in 5 categories.^22)^ The extent of viable residual carcinoma at the primary site was assessed semiquantitatively, based on the percentage of viable residual carcinoma relative to the macroscopically identifiable tumor bed, as evaluated histopathologically. The proportion of viable residual tumor cells within the cancerous tissue was evaluated as follows: Grade 3, no viable residual tumor cells (pCR); Grade 2, residual tumor cells accounting for less than one-third of the cancerous tissue; Grade 1b, residual tumor cells accounting for one-third to two-thirds of the cancerous tissue; Grade 1a, residual tumor cells accounting for more than two-thirds of the cancerous tissue; and Grade 0, no significant response to neoadjuvant chemotherapy.

### Statistical analysis

All statistical analyses were performed using IBM SPSS Advanced Statistics version 29.0 (IBM Corp., Armonk, NY, USA). Categorical variables were compared using the χ^2^ test or Fisher’s exact test, and continuous variables were analyzed using the Mann–Whitney U test. DFS and overall survival (OS) rates were calculated using the Kaplan–Meier method, and the Kaplan–Meier curves were visualized using GraphPad Prism version 10. DFS was defined as the time from the date of surgery to the first relapse of cancer or death from any cause, while OS was defined as the time from the date of surgery to death from any cause. Differences were considered significant at P-value <0.05. Values are expressed as medians.

To minimize baseline selection bias between patients who received adjuvant nivolumab and those who did not, propensity score matching (PSM) was conducted. The propensity scores were calculated using a logistic regression model incorporating clinically relevant covariates, including age and ypStage. Patients in the nivolumab group were matched 1:1 to those in the non-nivolumab group using the nearest-neighbor method without replacement, with a caliper width of 0.1 of the standard deviation of the logit of the propensity score.^23)^ After matching, the balance between groups was assessed using P-value.

Univariable and multivariable Cox proportional hazards regression analyses were performed to identify prognostic factors for DFS and OS. HRs and 95% CIs were calculated. Variables with a P-value <0.05 in the univariable analysis were included in the multivariable model. Adjuvant nivolumab was additionally entered into the multivariable model as a clinically relevant variable regardless of its significance in the univariable analysis.

Exploratory subgroup analyses for DFS were performed in selected high-risk pathological subgroups, including patients with ypStage IV, ypT3, and ypN1 disease. These subgroups were chosen based on their pathological features associated with a high risk of recurrence. HRs and 95% CIs were calculated for each subgroup. Given the limited sample size within each subgroup, these analyses were considered exploratory and hypothesis-generating. No adjustment for multiple comparisons was performed.

## Result

### Preoperative patient characteristics and surgical outcomes

A total of 259 patients underwent esophagectomy for esophageal cancer at the Department of Esophageal and Gastroenterological Surgery, Juntendo University Hospital. Among these, 224 had histologically confirmed squamous cell carcinoma, and 175 received neoadjuvant therapy, while 12 were excluded because of neoadjuvant ICI, 5 because of R1 resection, and 2 because of postoperative adjuvant therapy other than nivolumab. Of the remaining 156 patients, 16 achieved pathological complete remission, and 35 patients who received nCRT were excluded as well.

Finally, 105 patients with ESCC who underwent radical esophagectomy and exhibited an incomplete pathological response were enrolled in our study. Of these, 17 patients who received adjuvant nivolumab were assigned to the nivolumab group, and the other 88 patients who underwent observation alone were assigned to the non-nivolumab group.

The baseline clinicopathological characteristics are summarized in **Table 1**. In the whole cohort, the nivolumab group had more advanced disease, with significantly more cN2-3 disease (P = 0.044) and cM1 disease (P = 0.023). As for cT, there was no difference between both groups (P = 1.0). In the matched cohort, 16 patients were included in each group, and the baseline characteristics were well balanced. The P-values for cT and cN were both 1.000, suggesting that close balance was achieved after matching.

**Table 1:** Patient’s baseline characteristics before and after PSM

	Whole cohort			Matched cohort		
	Non-nivolumab N = 88	Nivolumab N = 17	P-value	Non-nivolumab N = 16	Nivolumab N = 16	P-value
Characteristics						
Age, years, mean ± SD	67.1 ± 10.52	64.8 ± 9.59	0.391	67.1 ± 10.4	65.8 ± 8.8	0.703
Sex, n (%)			0.348			0.600
Male	66 (75.0)	15 (88.2)		13 (81.3)	15 (93.8)	
Female	22 (25.0)	2 (11.8)		3 (18.8)	1 (6.3)	
cT, n (%)			1.000			1.000
1	4 (4.5)	0		1 (6.3)	0	
2	7 (8.0)	1 (5.9)		0	1 (6.3)	
3	73 (83.0)	16 (94.1)		15 (93.8)	15 (93.8)	
4	4 (4.5)	0		0	0	
cN, n (%)			0.044			1.000
0	30 (34.1)	3 (17.6)		4 (25.0)	3 (18.8)	
1	50 (56.8)	9 (52.9)		9 (56.3)	9 (56.3)	
2	8 (9.1)	4 (23.5)		3 (18.8)	3 (18.8)	
3	0	1 (5.9)		0	1 (6.3)	
cM, n (%)			0.023			0.458
0	72 (81.8)	9 (52.9)		12 (75.0)	9 (56.3)	
1	16 (18.2)	8 (47.1)		4 (25.0)	7 (43.8)	
cStage, n (%)			0.128			0.654
I	2 (2.3)	0		0	0	
II	27 (30.7)	3 (17.6)		4 (25.0)	3 (18.8)	
III	42 (47.7)	6 (35.3)		8 (50.0)	6 (37.5)	
IV	17 (19.3)	8 (47.1)		4 (25.0)	7 (43.8)	
Neoadjuvant chemotherapy, n (%)			0.685			0.600
DCF	77 (87.5)	16 (94.1)		13 (81.3)	15 (93.8)	
FP	11 (12.5)	1 (5.9)		3 (18.8)	1 (6.3)	

cT, clinical T category; cN, clinical N category; cM, clinical M category; cStage, clinical stage; DCF, docetaxel, cisplatin, and 5-fluorouracil; FP, cisplatin and 5-fluorouracil; PSM, propensity score matching

### Surgical outcomes and postoperative pathological findings

Postoperative complications are summarized in **Table 2**. The incidences of postoperative complications were comparable between the groups both before and after PSM. Before matching, recurrent laryngeal nerve palsy was the most frequent event in both groups. After matching, pneumonia was the most frequent event in the non-nivolumab group, while recurrent laryngeal nerve palsy was the most frequent event in the nivolumab group.

**Table 2:** A comparison of postoperative complications before and after PSM

Factor	Whole cohort			Matched cohort		
	Non-nivolumab (N = 88)	Nivolumab (N = 17)	P-value	Non-nivolumab (N = 16)	Nivolumab (N = 16)	P-value
Anastomotic leak, n (%)	11 (12.5)	2 (11.8)	1.000	2 (12.5)	2 (12.5)	1.000
Anastomotic stricture, n (%)	8 (9.1)	1 (5.9)	1.000	1 (6.3)	1 (6.3)	1.000
Pneumonia, n (%)	13 (14.8)	0	0.121	3 (18.8)	0	0.226
Recurrent laryngeal nerve palsy, n (%)	14 (15.9)	5 (29.4)	0.187	2 (12.5)	5 (31.3)	0.394
Chylothorax, n (%)	7 (8.0)	2 (11.8)	0.636	1 (6.3)	2 (12.5)	1.000
Reoperation, n (%)	7 (8.0)	1 (5.9)	1.000	0	1 (6.3)	1.000

PSM, propensity score matching

Postoperative pathological findings are summarized in **Table 3**. In the whole cohort, ypT and ypN were comparable between the groups, whereas ypM and ypStage differed significantly, reflecting a higher proportion of ypM1 and ypStage IVb disease (both P <0.001) in the nivolumab group. After PSM, particularly close balance in ypN, ypM, and ypStage was reached (P = 0.968, P = 1.000, and P = 0.938, respectively).

**Table 3:** Postoperative pathological findings before and after PSM

	Whole cohort			Matched cohort		
	Non-nivolumab N = 88	Nivolumab N = 17	P-value	Non-nivolumab N = 16	Nivolumab N = 16	P-value
Pathological findings						
ypT, n (%)			0.158			0.690
0	1 (1.1)	2 (11.8)		0	1 (6.3)	
1a	13 (14.8)	1 (5.9)		2 (12.5)	1 (6.3)	
1b	20 (22.7)	4 (23.5)		2 (12.5)	4 (25.0)	
2	25 (28.4)	3 (17.6)		3 (18.8)	3 (18.8)	
3	27 (30.7)	7 (41.2)		9 (56.3)	7 (43.8)	
4a	2 (2.3)	0		0	0	
ypN, n (%)			0.051			0.968
0	44 (50.0)	4 (23.5)		5 (31.3)	4 (25.0)	
1	30 (34.1)	6 (35.3)		6 (37.5)	6 (37.5)	
2	12 (13.6)	5 (29.4)		3 (18.8)	4 (25.0)	
3	2 (2.3)	2 (11.8)		2 (12.5)	2 (12.5)	
ypM, n (%)			<0.001			1
0	81 (92.0)	9 (52.9)		9 (56.3)	9 (56.3)	
1	7 (8.0)	8 (47.1)		7 (43.8)	7 (43.8)	
ypStage, n (%)			<0.001			0.938
I	32 (36.4)	1 (5.9)		1 (6.3)	1 (6.3)	
II	8 (9.1)	2 (11.8)		1 (6.3)	2 (12.5)	
III	41 (46.6)	6 (35.3)		7 (43.8)	6 (37.5)	
IV	7 (8.0)	8 (47.1)		7 (43.8)	7 (43.8)	

ypT, post-neoadjuvant pathological T category; ypN, post-neoadjuvant pathological N category; ypM, post-neoadjuvant pathological M category; ypStage, post-neoadjuvant pathological stage; PSM, propensity score matching

### Treatment outcomes of adjuvant nivolumab therapy: irAEs

Patients who received adjuvant nivolumab completed a median of 10 treatment cycles. irAEs occurred in 2 patients, including 1 case of adrenal insufficiency (at the seventh treatment cycle) and 1 case of hypothyroidism (at the fifth treatment cycle). Both irAEs were grade 3 and required therapeutic intervention, but no treatment-related mortality was observed. The summary of adjuvant nivolumab treatment and irAEs is shown in **Table 4**. The first case was a man in his 70s who developed adrenal insufficiency during the seventh cycle, and his symptoms resolved following corticosteroid administration. The second case was a woman in her 50s who experienced hypoglycemia and liver dysfunction during the fifth cycle and was ultimately diagnosed with grade 3 hypothyroidism. Her symptoms improved with thyroid hormone replacement therapy.

**Table 4:** Summary of adjuvant nivolumab treatment and irAEs

Variable	Nivolumab group (N = 17)
Total cycles, median (range)	10 (1–19)
Completed planned 1-year treatment, n (%)	11 (64.7%)
Completed ≥10 cycles, n (%)	9 (52.9%)
Patients with irAEs, n (%)	2 (11.8%)
Cycle at onset of irAEs, median (range)	6 (5–7)
Type of irAEs, n	Adrenal insufficiency (1), hypothyroidism (1)
Severity (CTCAE grade)	3
Management	Steroids (1), thyroid hormone (1)

irAEs, immune-related adverse events; CTCAE, National Cancer Institute Common Terminology Criteria for Adverse Events

### Patterns of recurrence

Among the 105 patients, recurrence was observed in 33 patients in the whole cohort, including 27 in the non-nivolumab group and 6 in the nivolumab group. No statistically significant differences were observed in total recurrence, locoregional recurrence, or distant recurrence between the 2 groups in the whole cohort. In the matched cohort, recurrence occurred in 9 patients in the non-nivolumab group and 5 patients in the nivolumab group. Locoregional recurrence was numerically less frequent in the nivolumab group, whereas distant recurrence was comparable between the 2 groups. No statistically significant differences in recurrence patterns were observed in the matched cohort. Details of recurrence patterns are summarized in **Table 5**.

**Table 5:** Patterns of recurrence

Patterns of recurrence	Whole cohort			Matched cohort		
	Non-nivolumab N = 88	Nivolumab N = 17	P-value	Non-nivolumab N = 16	Nivolumab N = 16	P-value
Total recurrence, n (%)	27 (30.7)	6 (35.3)	0.778	9 (56.3)	5 (31.3)	0.285
Locoregional recurrences	21 (23.9)	2 (11.8)	0.351	7 (43.8)	2 (12.5)	0.113
Regional lymph node	18 (20.5)	2 (11.8)		6 (37.5)	2 (12.5)	
Local	5 (5.7)	0		2 (12.5)	0	
Distant recurrences	15 (17.0)	6 (35.3)	0.102	5 (31.3)	5 (31.3)	1.000
Distant lymph node	7 (8.0)	3 (17.6)		2 (12.5)	2 (12.5)	
Liver	4 (4.5)	1 (5.9)		3 (18.8)	1 (6.3)	
Lung	1 (1.1)	1 (5.9)		0	1 (6.3)	
Bone	2 (2.3)	1 (5.9)		1 (6.3)	1 (6.3)	
Any dissemination	6 (6.8)	2 (11.8)		0	2 (12.5)	

### Survival analysis

During a median follow-up of 26.8 months, a total of 18 deaths were observed, including 2 in the nivolumab group. Among these, 1 death was due to another disease and 1 was due to chronic renal failure, whereas the remaining deaths were related to the primary disease.

**Fig. 1:**
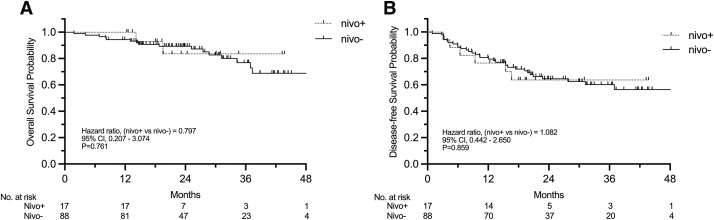
Kaplan–Meier curves for OS and DFS in the entire cohort before PSM. OS (**A**) and DFS (**B**) were compared between the nivolumab and non-nivolumab groups before propensity score matching. OS, overall survival; DFS, disease-free survival; PSM, propensity score matching

In the whole cohort, the 1-year OS rate was 100.0% in the nivolumab group and 94.3% in the non-nivolumab group. The nivolumab group showed a transient downward shift in the mid-follow-up period, but OS did not differ significantly between the groups (P = 0.761). The OS curves are presented in **Fig. 1A**. The 1-year DFS rate was 76.5% in the nivolumab group and 80.6% in the non-nivolumab group (P = 0.859). The DFS curves were largely overlapping between the 2 groups throughout follow-up, and no significant difference was observed. The DFS curves are presented in **Fig. 1B**.

**Fig. 2:**
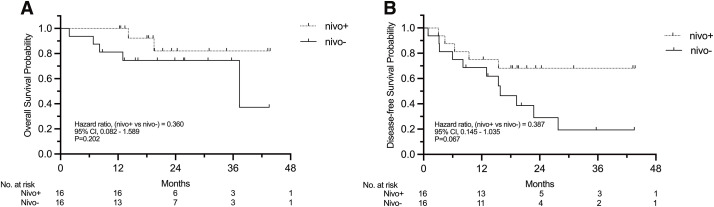
Kaplan–Meier curves for OS and DFS after PSM. OS (**A**) and DFS (**B**) were compared between the nivolumab and non-nivolumab groups after PSM. OS, overall survival; DFS, disease-free survival; PSM, propensity score matching

In the matched cohort, the 1-year OS rates were 100.0% and 81.3%, respectively. The OS curves for the matched cohort are presented in **Fig. 2A**. OS remained comparable between the nivolumab and non-nivolumab groups, with no statistically significant separation of the Kaplan–Meier curves (P = 0.202). The DFS curves for the matched cohort are presented in **Fig. 2B**. The 1-year DFS rate was 75% in the nivolumab group and 68.8% in the control group, while no significant difference was observed in DFS following adjustment for baseline characteristics (P = 0.067). Although the difference did not reach statistical significance, the DFS trend showed an improvement. The 3-year DFS rates were 68.2% and 19.3%, respectively. The nivolumab group showed a prolonged DFS trend, as the Kaplan–Meier curve is higher during long-term follow-up, which means a slower accumulation of events compared with the control group.

To further identify independent prognostic factors for OS/DFS and adjust for potential confounding, we performed univariable and multivariable Cox proportional hazards analyses shown, as in **Table 6** (OS) and **Table 7** (DFS). In the univariable analysis for OS, there were no significantly prognostic factors in clinical variables. In the multivariable analysis, advanced ypT stage (ypT3–4a) (P = 0.011) and advanced ypN stage (ypN2–3) (P = 0.002) remained independently associated with worse OS. Adjuvant nivolumab was not a significantly prognostic factor in OS (HR 0.505 [0.112–2.283], P = 0.375) after adjustment for ypT and ypN. In the univariable analysis for DFS, adjuvant nivolumab showed a numerically higher hazard of DFS compared with non-nivolumab (HR 1.082 [0.452–2.593], P = 0.859). In the multivariable analysis, ypT0–2 and ypN0–1 remained independently associated with better DFS (HR 0.351 [0.181–0.683], P = 0.002; HR 0.237 [0.116–0.484], P <0.001, respectively). The HR for adjuvant nivolumab shifted from above one in the univariable analysis to below one after adjustment for ypT and ypN (HR 0.602 [0.240–1.511], P = 0.280), suggesting a nonsignificant trend toward reduced DFS risk.

**Table 6:** Univariate and multivariate Cox regression analyses for the OS

Factor	Category	Univariable analysis		Multivariable analysis	
		HR (95% CI)	P-value	HR (95% CI)	P-value
Age	Per year increase	1.048 (0.991–1.107)	0.099		
Sex	Female vs. male	0.610 (0.176–2.114)	0.436		
cT	cT1-2 vs. cT3-4	1.200 (0.345–4.167)	0.774		
cN	cN0 vs. cN1-3	0.972 (0.362–2.604)	0.954		
cStage	cStage I–II vs. III–IV	1.314 (0.506–3.413)	0.575		
ypT	ypT0-2 vs. ypT3-4a	0.250 (0.097–0.648)	0.004	0.287 (0.109–0.752)	0.011
ypN	ypN0-1 vs. ypN2-3	0.163 (0.055–0.477)	<0.001	0.170 (0.056–0.518)	0.002
ypStage	ypStage I–II vs. III–IVb	0.423 (0.149–1.200)	0.106		
Adjuvant nivolumab	Nivo+ vs. Nivo−	0.796 (0.182–3.477)	0.762	0.505 (0.112–2.283)	0.375

cT, clinical T category; cN, clinical N category; cStage, clinical stage; ypT, post-neoadjuvant pathological T category; ypN, post-neoadjuvant pathological N category; ypStage, post-neoadjuvant pathological stage; HR, hazard ratio; CI, confidence interval; OS, overall survival

**Table 7:** Univariate and multivariate Cox regression analyses for the DFS

Factor	Category	Univariable analysis		Multivariable analysis	
		HR (95% CI)	P-value	HR (95% CI)	P-value
Age	Per year increase	1.022 (0.987–1.059)	0.211		
Sex	Female vs. male	0.771 (0.339–1.753)	0.535		
cT	cT1-2 vs. cT3-4	0.749 (0.264–2.123)	0.587		
cN	cN0 vs. cN1-3	0.514 (0.235–1.122)	0.095		
cStage	cStage I–II vs. III–IV	0.538 (0.246–1.176)	0.121		
ypT	ypT0-2 vs. ypT3-4a	0.342 (0.180–0.650)	0.001	0.351 (0.181–0.683)	0.002
ypN	ypN0-1 vs. ypN2-3	0.240 (0.121–0.475)	<0.001	0.237 (0.116–0.484)	<0.001
ypStage	ypStage I–II vs. III–IVb	0.508 (0.251–1.027)	0.059		
Postoperative complication	Yes vs. no	1.045 (0.553–1.977)	0.892		
Adjuvant nivolumab	Nivo+ vs. Nivo−	1.082 (0.452–2.593)	0.859	0.602 (0.240–1.511)	0.280

cT, clinical depth of tumor invasion; cN, clinical lymph node metastasis; cStage, clinical Stage; ypT, post-neoadjuvant pathological depth of tumor invasion; ypN, post-neoadjuvant pathological lymph node metastasis; ypStage, post-neoadjuvant pathological stage; HR, hazard ratio; CI, confidence interval; DFS, disease-free survival

**Fig. 3:**
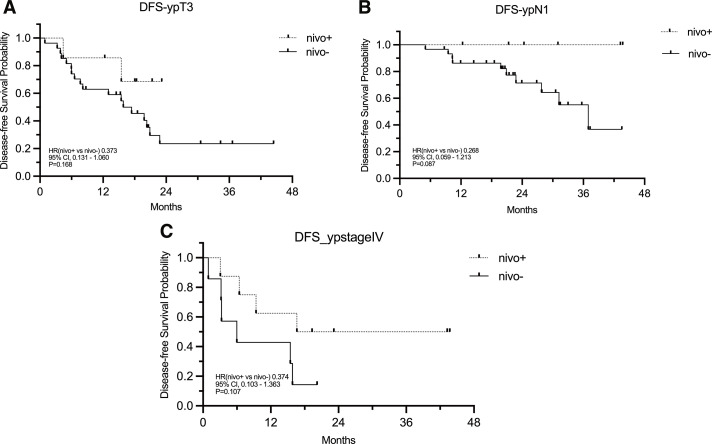
Kaplan–Meier curves for DFS in selected pathological subgroups. DFS was compared between the nivolumab and non-nivolumab groups in patients with ypT3 (**A**), ypN1 (**B**), and ypStage IV (**C**). DFS, disease-free survival

Based on the multivariable findings, we further assessed whether the association between adjuvant nivolumab and survival outcomes was consistent across post-neoadjuvant pathological stages. Exploratory subgroup analyses of DFS were performed across ypT, ypN, and ypStage. In the presented high-risk pathological subgroups, adjuvant nivolumab was consistently associated with a favorable DFS trend compared with no nivolumab, including in patients with ypT3 (HR 0.48 [0.17–1.36], P = 0.168), ypN1 (HR 0.27 [0.06–1.21], P = 0.087), and ypStage IV (HR 0.34 [0.09–1.26], P = 0.107). The DFS results in the selected subgroups are shown in **Figs. 3A**–**3C**. Although none of these subgroup comparisons reached statistical significance, all 3 analyses showed a consistent direction favoring adjuvant nivolumab.

## Discussion

The survival impact of adjuvant immunotherapy for esophageal cancer has been defined largely by the CheckMate 577 trial, which demonstrated a significant improvement in DFS with adjuvant nivolumab in patients with an incomplete pathological disease following nCRT.^2)^ In this global, randomized phase III trial, nivolumab almost doubled median DFS compared with the control group, establishing immune checkpoint inhibition as a new standard of care. The majority of enrolled patients had ESCC, which is the histologic subtype most prevalent in Japan. However, the efficacy estimated within real-world Asian ESCC populations remained less powerful due to the inevitable differences in disease biology, treatment patterns, and patient characteristics. Most importantly, the efficacy and safety of adjuvant nivolumab in patients receiving neoadjuvant chemotherapy remain unexplored. In our study, we specifically selected those patients who underwent neoadjuvant chemotherapy, and our treatment model is consistent with the design of the CheckMate 577 trial.

Several observational studies have subsequently evaluated adjuvant nivolumab in real-world clinical settings. Reports from other Japanese centers have shown trends toward improved DFS, particularly in patients with high-risk pathological features, although the magnitude of benefit has varied.^24,25)^ Early Japanese observational cohorts indicate that adjuvant nivolumab is feasible and exhibits recurrence-suppressing potential, particularly in patients with a partial response to neoadjuvant therapy. In a Japanese cohort of resected ESCC treated with neoadjuvant chemotherapy, Sugase et al. reported that adjuvant nivolumab was more likely to prolong 1-year recurrence-free survival, suggesting a clinically meaningful signal for recurrence suppression in routine practice.^24)^ Building on this, a subsequent study by Sugase et al. evaluated a single-center retrospective cohort of locally advanced ESCC treated with neoadjuvant DCF followed by radical esophagectomy, in which adjuvant nivolumab was associated with improved DFS and OS after PSM, with the greatest benefit suggested in patients with more advanced pathological disease (e.g., ypT3/ypN2 and ypStage III).^25)^ Similarly, Koga et al. concluded in a Japanese single-center cohort that adjuvant nivolumab following neoadjuvant chemotherapy and esophagectomy may improve DFS, and they additionally explored the relationship between irAEs and survival outcomes, which had the same patient selection as our study.^10)^

Under this background, we assessed DFS and OS in patients with ESCC who underwent neoadjuvant chemotherapy, comparing those who received adjuvant nivolumab with those managed by observation alone. Our analysis showed that adjuvant nivolumab was not associated with statistically significant improvements in DFS or OS in the overall cohort. After PSM, both DFS and OS showed favorable trends in the nivolumab group, with visually evident late separation of the survival curves, although statistical significance was not reached. In selected pathological subgroups, particularly those with high-risk residual disease, the DFS curves showed more favorable separation and relatively lower P-values, although these analyses remained exploratory and underpowered. Although the overall distribution of recurrence patterns was comparable between groups, lymph node involvement remained the predominant pattern of first relapse. The recurrence-pattern analysis suggests that adjuvant nivolumab may contribute to locoregional control. By contrast, distant relapse may reflect a greater systemic tumor burden, which may be less likely to be modified by postoperative immunotherapy alone in our small cohort. Interestingly, our interpretation is consistent with a Japanese real-world study.^25)^ In this Sugase’s study, adjuvant nivolumab was associated with reductions in both locoregional and distant recurrences, although the suppressive effect appeared to be greater for locoregional recurrences than for distant recurrences.

The discrepancy between the overall cohort and the matched cohort is likely attributable to patient selection bias in our study, as patients in the nivolumab group had more advanced disease both clinically and pathologically than those in the non-nivolumab group before matching. This imbalance may have arisen from our stronger recommendation of adjuvant nivolumab for patients with more advanced disease. After PSM, the baseline characteristics were better balanced, and the direction of the survival curves became more consistent with previous Japanese real-world studies. Although the differences in DFS and OS did not reach statistical significance, the favorable trend after matching supports the possibility that adjuvant nivolumab may be beneficial for appropriately selected patients with residual disease after neoadjuvant chemotherapy. Similarly, although adjuvant nivolumab was not statistically significant in the multivariable Cox regression analysis, the direction of the HR changed after adjustment, shifting toward a favorable effect. This finding suggests that the lack of benefit in the unadjusted analysis may have been influenced by baseline imbalance. Consistently, subgroup analyses stratified by selected high-risk pathological stage demonstrated that the effect of adjuvant nivolumab may not be uniform across all patients. This pattern is consistent with previous Japanese reports suggesting that patients with more advanced pathological features may derive greater benefit from adjuvant nivolumab after neoadjuvant chemotherapy. Therefore, rather than indicating a lack of efficacy, our subgroup findings suggest that the potential benefit of adjuvant nivolumab may be concentrated in specific high-risk populations.

Although our findings did not demonstrate statistically significant survival improvement, they are not contradictory to previous Japanese reports. Rather, the favorable post-matching trends in DFS and OS, together with the late separation of the survival curves, are consistent with prior real-world studies. Three possible explanations may be considered for the absence of statistical significance in our cohort. First, the sample size and number of survival events were limited, particularly after PSM, reducing the power to detect moderate treatment effects. Second, the disease burden in our nivolumab group was extremely advanced, with nearly half of the patients classified as pStage IVB, which may have persisted even after adjustment. Third, the therapeutic benefit of adjuvant nivolumab may depend on pathological risk profiles, treatment duration, and immune-related factors, including the occurrence and management of irAEs. Although these findings suggest a possible survival benefit of adjuvant nivolumab after adjustment for baseline imbalance, the limited sample size and number of events mean that this finding should be interpreted cautiously. The ongoing randomized controlled trial JCOG2206 is expected to provide more definitive evidence regarding the role of adjuvant nivolumab after neoadjuvant chemotherapy.

This study has several limitations that must be acknowledged. First, its retrospective, single-center design introduces the possibility of confounding, even though PSM was performed. Second, the modest cohort size limits the generalizability of possible findings and restricts subgroup analysis. Third, for some immunohistochemistry markers, such as PD-L1 expression, our center does not conduct them routinely, so there may not be any data analysis on the prediction and prognosis of the safety and efficacy of postoperative nivolumab. Finally, heterogeneity in recurrence definitions and the relatively short prognostic follow-up period may have introduced detection bias, thereby potentially underestimating event rates. Further multicenter investigations with larger sample sizes, longer follow-up, and biomarker-based stratification are needed to validate these observations and to identify patients who are most likely to benefit from adjuvant nivolumab after neoadjuvant chemotherapy.

## Conclusion

In patients with ESCC who achieved an incomplete pathological response after neoadjuvant chemotherapy, adjuvant nivolumab was associated with a favorable trend in both DFS and OS compared with those who did not receive nivolumab in this single-center retrospective cohort, with late survival separation favoring the nivolumab group, although statistical significance was not reached. AEs were generally manageable and consistent with the known safety profile of PD-1 blockade. Given the limited sample size and potential confounding in this non-randomized setting, larger multicenter studies with longer follow-up and biomarker evaluation are needed to clarify the real-world effectiveness of adjuvant nivolumab and to refine its optimal indications after neoadjuvant chemotherapy and esophagectomy.

## Declarations

### Data availability

The data that support the findings of this study are not publicly available due to privacy restrictions, but are available from the corresponding author upon reasonable request.
